# Feasibility and reliability of the Spondyloarthritis Research Consortium of Canada sacroiliac joint inflammation score in children

**DOI:** 10.1186/s13075-018-1543-x

**Published:** 2018-03-22

**Authors:** Pamela F. Weiss, Walter P. Maksymowych, Robert G. Lambert, Jacob L. Jaremko, David M. Biko, Joel Paschke, Timothy G. Brandon, Rui Xiao, Nancy A. Chauvin

**Affiliations:** 1 Department of Pediatrics, Division of Rheumatology, Children’s Hospital of Philadelphia, Philadelphia, PA USA; 2 Department of Radiology, Children’s Hospital of Philadelphia, Philadelphia, PA USA; 3 Center for Pediatric Clinical Effectiveness (CPCE), Children’s Hospital of Philadelphia, Philadelphia, PA USA; 4 Center for Clinical Epidemiology and Biostatistics, Perelman School of Medicine at the University of Pennsylvania, Philadelphia, PA USA; 5 Department of Medicine, University of Alberta, Edmonton, AB Canada; 6 Department of Radiology and Diagnostic Imaging, University of Alberta, Edmonton, AB Canada; 7 Canadian Research and Education (CaRE) Arthritis Organization, Edmonton, AB Canada; 8 The Children’s Hospital of Philadelphia, Roberts Center for Pediatric Research, 2716 South Street, Room 11121, Philadelphia, PA 19146 USA

**Keywords:** Imaging, Spondyloarthritis, Ankylosing spondylitis, Juvenile idiopathic arthritis, Outcomes

## Abstract

**Background:**

Published methods for quantification of magnetic resonance imaging (MRI) evidence of inflammation in the sacroiliac joint lack validation in pediatric populations. We evaluated the reliability and construct validity of the Spondyloarthritis Research Consortium of Canada (SPARCC) sacroiliac joint inflammation score (SIS) in children with suspected or confirmed juvenile spondyloarthritis (JSpA).

**Methods:**

The SPARCC SIS measures the presence, depth, and intensity of bone marrow inflammation on MRI through the cartilaginous part of the joint. Six readers blinded to clinical details except age, participated in two reading exercises, each preceded by a calibration exercise. Inter-observer reliability was assessed using intraclass correlation coefficients (ICCs) and for pre-specified acceptable reliability the inraclass correlation coefficient (ICC) was > 0.8.

**Results:**

The SPARCC SIS had face validity and was feasible to score in pediatric cases in both reading exercises. Cases were mostly male (64%) and the median age at the time of imaging was 14.9 years. After calibration, the median ICC across all readers for the SIS total score was 0.81 (IQR 0.71–0.89). SPARCC SIS had weak correlation with disease activity (DA) as measured by the JSpADA (*r* = − 0.12) but discriminated significantly between those with and without elevated C-reactive protein (*p* = 0.03).

**Conclusion:**

The SPARCC SIS was feasible to score and had acceptable reliability in children. The ICC improved with additional calibration and reading exercises, for both experienced and inexperienced readers.

## Background

Children with spondyloarthritis (SpA) and axial arthritis are at risk of progression to ankylosing spondylitis, an inflammatory disease that causes joint fusion and leads to permanent functional impairment. While there are similarities between juvenile and adult SpA, there are distinct phenotypic differences that warrant specific focus and clinical trials in juvenile disease [1–5]. In order to assess the efficacy and effectiveness of medications for SpA and axial disease in critically needed clinical trials, we need an objective measure to quantify response in the pediatric sacroiliac joint.

There are five Food and Drug Administration (FDA)-approved anti-TNF drugs for ankylosing spondylitis in adults and many more phase III trials underway. There has only been one trial of biologic agents in children with axial arthritis [6]. This trial had several limitations leading to findings that were not statistically significant for the primary outcome. Noteworthy limitations were that imaging was not part of the protocol and the primary outcome was achievement of an Assessment of SpA International Society (ASAS) working group 40 response criteria [7], which has never been validated in children. Despite the negative results, most pediatric rheumatologists believe TNF inhibitors are effective. This trial experience underscores the importance of identifying clinically meaningful and responsive measures of inflammation in the pediatric sacroiliac joint.

Validated measures to objectively assess inflammation in the adult sacroiliac joint include the modified Berlin magnetic resonance imaging (MRI) method [8] and the SPondylo Arthritis Research Consortium of Canada (SPARCC) Sacroiliac joint Inflammation Score (SIS) [9]. The modified Berlin method grades the proportion of bone marrow edema in each sacroiliac joint according to the percentage of each joint quadrant (grade 1, < 33%; grade 2, ≥ 33–66%; grade 3, > 66%) affected by edema so that the total score for both SIJs ranges from 0 to 24. The SPARCC SIS measures the presence, depth, and intensity of bone marrow inflammation in each sacroiliac joint quadrant in consecutive semi-coronal slices through the joint. The SPARCC SIS has been used successfully in clinical trials of biologic agents in adults with SpA and axial disease [10–14]. Improvements in inflammation on MRI were significantly correlated with change in several clinical measures including composite disease activity measures and C-reactive protein levels [11].

Identification of useful measures of inflammation in the pediatric sacroiliac joint will translate into improved care algorithms and increased opportunities for labeling of emerging targeted medications for these children. We evaluated the reliability and construct validity of the SPARCC SIS in children with suspected or confirmed juvenile SpA.

## Methods

This study protocols were reviewed and approved by the Children’s Hospital of Philadelphia Committee for the Protection of Human Subjects (16–012641 and 17–013883). Waivers of consent and HIPAA authorization were granted as the procedures represented minimal risk to the subjects and did not adversely affect the rights and welfare of the subjects.

Cases were children with suspected or confirmed SpA who had imaging performed at the Children’s Hospital of Philadelphia (Philadelphia, PA, USA) or Stollery Children’s Hospital (Edmonton, Alberta, Canada). All images were de-identified prior to scoring and demographics were abstracted from the clinical chart. The imaging protocols at the two institutions varied slightly and minor modifications of the imaging protocols over the time period were noted. The SPARCC SIS scores, however, only require two specific sequences, which were uniformly performed on all included studies with the T1-weighted and short-tau inversion recovery (STIR) coronal oblique sequences. One of the advantages of the SPARCC tool is that as long as these two sequences are present, subtle differences in MRI procedure amongst institutions do not impact its applicability.

**Fig. 1:**
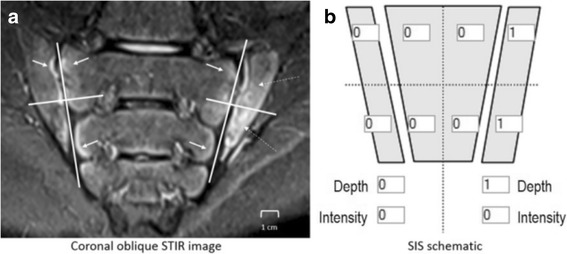
Sample slice and scoring methods for sacroiliac joint inflammation score (SIS). **a** Coronal oblique short-tau inversion recovery (STIR) image of the sacroiliac joints shows each joint divided into four quadrants. There is normal bright subchondral signal along both sides of the right sacroiliac joint and along the periarticular region of the left sacrum (solid arrows) in this skeletally immature patient. On the left, there is abnormal signal consistent with bone marrow edema within the periarticular region of the left ilium in both the upper and lower quadrants (dashed arrows). **b** Scoring schematic demonstrates a “1” within the left iliac bone in both the upper and lower quadrants representing bone marrow edema. An additional score of “1” is given as the depth of the bone marrow edema extends > 1 cm from the articular surface. The patient received a total score of “3” for this image slice

The SPARCC SIS assesses the presence, depth and intensity of bone marrow edema on consecutive fluid sensitive coronal oblique slices through the cartilaginous part of the joint (Table 1). Six consecutive semi-coronal slices through the cartilaginous portion of the joint are scored for bone marrow edema (BME; Fig. 1). The presence or absence of edema is scored for each joint quadrant (total score per slice 0–8). The presence or absence of bone marrow signal intensity (present if intensity is the same or greater than the presacral veins) and depth ≥1 cm are scored dichotomously for each sacroiliac joint. T1-weighted and STIR sequences are viewed simultaneously to help the reader define the anatomy but only STIR sequences are scored. The total score ranges from 0 to 72 with higher scores indicating more inflammation. We completed two calibration modules and two reading exercises over the course of approximately 18 months.

**Table 1:** Scoring system used to evaluate six consecutive semi-coronal magnetic resonance imaging slices (total score range 0–72)

Feature	Definition	Score range per slice	Maximum score
Bone marrow edema	Hyperintense signal on short-tau inversion recovery	Score 4 quadrants in each sacroiliac joint 0/1, range per slice is 0–8	48
Bone marrow intensity	Hyperintensity of marrow edema using the presacral veins as reference	Score each sacroiliac joint 0/1, range per slice is 0–2	12
Bone marrow depth	Homogeneous increase of marrow signal ≥1 cm from the articular surface within either sacroiliac joint	Score each sacroiliac joint 0/1, range per slice is 0–2	12

All training and scoring exercises were performed within a web-based environment (CaREArthritis.com) by six readers (three pediatric radiologists, one pediatric rheumatologist, one adult radiologist and one adult rheumatologist). Two of the readers were SPARCC SIS developers (WM and RL). Before completing reading exercise 1, all readers reviewed a pediatric PowerPoint training module that included scoring instructions and examples of bone marrow edema and bright subchondral signal on STIR scans that may be easily confused with inflammation. The reference for normal bone marrow signal intensity in children was the iliac crests, edges of the vertebral bodies, tri-radiate cartilage and ischial pubic synchondrosis. Each reader, blinded to clinical details except age, subsequently used the online viewing and scoring system to score 30 digital imaging and communication in medicine (DICOM)-based studies that included semi-coronal T1-weighted and STIR sequences. The data for reading exercises 1 and 2 were based on data from a single time point.

After reading exercise 1, all readers, except WM and RL (SPARCC SIS developers), completed an interactive calibration module comprising 40 adult cases, each with studies of T1-weighted and STIR scans from baseline and 2 years after initiation of TNF inhibitor therapy (https://www.carearthritis.com/mriportal/sparccsij/index/). Each study was scored in pairs, blinded to time point. Readers received instantaneous feedback on concordance/discordance of scoring with the expert readers (WM and RL) after scoring each individual semi-coronal slice in the first 20 cases. Feedback on the next 20 cases was provided after scoring the entire case. The intraclass correlation coefficient (ICC) for concordance with the expert readers was calculated after the first 20 cases were completed, again after the next 10 cases, and finally after all 40 cases had been scored. The *a priori* score deemed acceptable for this calibration was an ICC >0.8 for the total SPARCC SIS score. After the interactive training module, we conducted a second reading exercise based on DICOM images comprising T1-weighted and STIR scans from 30 pediatric studies, which were a mix of 15 studies from the first exercise and 15 new studies.

Face and content validity were established through discussion during the creation of the pediatric PowerPoint training module and initial calibration exercise. Face and content validity is demonstrated if the criteria are sensible on face value to physicians who utilize them in their field. Feasibility was assessed by asking the raters to estimate the time it took to score each case and to rate the ease with which it was performed.

Inter-observer reliability was assessed using ICC. The ICCs are presented as agreement for all readers together, SPARCC developers (*n* = 2), pediatric radiologists (*n* = 3) and rheumatologists (n = 2). We applied the pre-specified ICC target from the interactive adult calibration module to the interpretation of the results from the two reading exercises (> 0.8 for the total score).

Construct validity was tested using Spearman’s rho, measuring correlation between the mean SPARCC SIS developers’ status scores from reading exercise 2 and disease activity as measured by the juvenile SpA disease activity (JSpADA) index (range 0–8) [15] and physician global disease activity assessment (visual analogue scale, range 0–10). The JSpADA index is a disease activity measure that is scored 0 to 8, with higher scores indicating more active disease. The components of the score are weighted equally and consist of the following measures: arthritis, enthesitis, patient pain assessment, inflammatory markers, morning stiffness, clinical sacroiliitis, uveitis and back mobility. Discrimination was tested by comparing the mean SPARCC SIS developers’ scores with laboratory values (C-reactive protein ≥1 or <1 mg/dl and erythrocyte sedimentation rate ≥21 or <21 mm/h) and patients with and without inflammatory back pain. Laboratory values were included if available +/− 60 days from the date of the MRI.

## Results

### Subjects

Demographic and clinical characteristics of the cases included in the two reading exercises are shown in Table 2. In total there were 45 unique cases from two tertiary care centers that were utilized for the two reading exercises. There were 29 (64%) male patients and median age at the time of imaging was 14.9 years (IQR 12.7–16.5, range 6.8–18.7 years). At the time of imaging, most children and adolescents had minimal peripheral disease activity (median active joint and tender entheses counts of 0) but moderate self-reported pain (median 4) and self-reported and physician-reported disease activity (3 and 3, respectively).

**Table 2:** Case characteristics

	Number	Frequency (%)/median (IQR)
Age (years)	45	14.9 (12.7–16.5)
Sex-male	45	29 (64.4%)
HLA-B27+	39	25 (64.1%)
Race	39	
Caucasian		32 (82.1%)
African American		5 (12.8%)
Other		2 (5.1%)
Active joint count	36	0 (0–1)
Tender enthesis count	36	0 (0–5)
Patient-reported pain^a^ (VAS; range 0–10)	32	3.8 (1–6)
Patient-reported disease activity (VAS; range 0–10)	31	3 (0.5–5.1)
Physician global disease activity (VAS; range 0–10)	31	3 (2–4)
CRP (≥1.0 mg/dL)	28	17 (60.7%)
ESR (≥21 mm/h)	29	12 (41.4%)

There were 45 unique cases assessed in the two reading exercises

*VAS* visual analogue scale, *CRP* C-reactive protein, *ESR* erythrocyte sedimentation rate

^a^General pain recorded on a visual analogue scale

### Feasibility and interobserver reliability

The SPARCC SIS had acceptable face validity and was feasible to score. The time required to assess a case for the presence, depth and intensity of lesions for a single case less than 15 min. All readers felt the online scoring platform was easy to use.

**Fig. 2:**
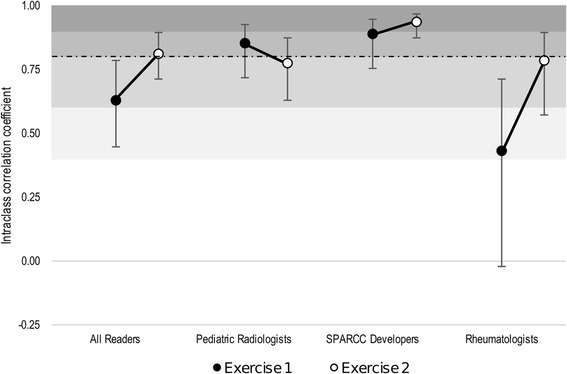
Intraclass correlation coefficients of the Spondyloarthritis Research Consortium of Canada (SPARCC) sacroiliac joint inflammation score across two exercises

In exercise 1, 140 (78%) studies read by all six readers had a SPARCC SIS ≥2 and the median SPARCC SIS was 14 (IQR 3–29). The ICCs for the SPARCC SIS total score from both reading exercises are shown in Fig. 2. In the first exercise, both the SPARCC developers and pediatric radiologists achieved the pre-specified target of > 0.8 for the total score. The ICC amongst all readers was 0.63 (IQR 0.45–0.78).

In between the first and second reading exercises, the four readers who were not SPARCC SIS developers (PW, JJ, DB and NC) completed an interactive calibration module comprising 40 adult cases. Three of the four readers rated the scoring platform for the calibration module as easy to use and the time to score each case ranged from 4 to 15 min. All four readers achieved an ICC status score >0.82 (range 0.82–0.99).

In exercise 2, 133 (74%) of the studies had a SPARCC SIS ≥2. Median SPARCC SIS was 7.5 (IQR 1–24.5). The ICC amongst all six readers surpassed the pre-specified target of 0.8. When assessed by different rater backgrounds, only the SPARCC developers achieved the pre-specified target of > 0.8 for the total score. The ICCs amongst pediatric radiologists and rheumatologists were 0.77 and 0.79, respectively (Fig. 2).

### Construct validity

Correlation between disease activity, as measured by the JSpADA index and physician global disease activity, and the SPARCC SIS developers’ mean scores for all unique patients was weak (*r* = − 0.12, *p* = 0.64 and *r* = 0.09, *p* = 0.65, respectively). The SPARCC SIS discriminated well between those children with and without elevated C-reactive protein (*p* = 0.03) but not between children with and without inflammatory back pain (*p* = 0.92).

## Discussion

Identification of reliable, objective, and quantifiable measures of response in children with axial disease is critical. Without measures to objectively quantify the severity of disease in the pediatric sacroiliac joint, assessment of interval change and response to medication in efficacy or effectiveness studies is not possible. This is the first evaluation of the feasibility and reliability of the SPARCC SIS in children. The SPARCC SIS was feasible to score and achieved the pre-specified target for acceptable reliability (ICC ≥0.8) in children. The ICC improved and surpassed the pre-specified acceptable reliability threshold with additional calibration and reading exercises, even for readers with limited experience. The SPARCC SIS did not have convergent validity with clinical measures of disease activity, highlighting that imaging and clinical evaluations provide complimentary but non-overlapping information. The SPARCC SIS did discriminate between those children with and without elevated C-reactive protein but did not discriminate between children with and without back pain.

Our findings indicate that readers with various levels of imaging experience can have their reading calibrated to acceptable levels of reliability using a relatively simple calibration module that provides iterative real-time feedback on the concordance/discordance of their scoring with the expert readers at the level of individual sacroiliac joint quadrants. This is promising as this means that radiologists (both adult and pediatric) from geographically isolated institutions can have their reading calibrated for the purposes of both efficacy and effectiveness studies. In the first reading exercise both the pediatric radiologists (*n* = 3) and SPARCC developers (*n* = 2) attained an ICC >0.8 yet the overall ICC was lower (0.63). The lower total group ICC was likely secondary to inclusion of an inexperienced reader (PW) and to systematic differences in the way in which the pediatric radiologists and the SPARCC developers approach evaluation of these studies. The subsequent calibration exercises were meant to reduce these betwee-group differences and heighten overall agreement or reliability, which they did. Interestingly, the ICC in the pediatric radiology group dropped slightly in the second reading exercise, which might signal that each of the pediatric radiologists adjusted his/her interpretations slightly differently and that perhaps with additional training the total ICC can improve even more. It would be ideal in the future to develop an interactive calibration module based solely on pediatric imaging studies.

In adults, the SPARCC SIS has been shown to significantly correlate with the magnitude of C-reactive protein and other measures of clinical disease activity [11] that have not been validated in children. We did not see correlation with disease activity measures, perhaps secondary to the relatively small sample size or the lack of validated specific measures of axial disease activity to use as reference standards. One measure specifically designed to assess clinical disease activity in juvenile SpA is the JSpADA index [15], but this is an assessment of overall SpA disease activity and axial disease activity accounts for only two of eight variables contributing to the score. No other validated juvenile arthritis disease activity tools assess axial disease activity. The SPARCC SIS did discriminate between those with and without elevated C-reactive protein. This is not entirely surprising given the results from the aforementioned adult studies [11] and the pediatric studies demonstrating increased predictive probability of sacroiliitis with elevated C-reactive protein [4]. Discrimination was not uniform across multiple clinical attributes as it did not discriminate those with and without back pain. This latter finding may be a problem unique to pediatric SpA in which back pain has been shown to be poorly correlated with the presence of axial arthritis in multiple studies [4, 16].

There are several limitations to our study that should be considered. First, the number of pediatric studies was limited, largely due to missing semi-coronal sequences which are essential for SPARCC scoring. The limited number of studies necessitated re-evaluation during the second exercise of some cases used in the first reading exercise. We do not believe using the same studies more than once impacted our results because the cases were not discussed amongst the readers after the first reading exercise and the two reading exercises were separated by approximately 15 months. The limited number of studies also means we could not assess the minimal detectable difference or change in scores; this will be a focus of future work. Second, the studies utilized in the reading and calibration exercises were primarily from only two North American hospitals. Data collection from only two hospitals frequently leads to overrepresentation of regional demographics and a cohort that may not be typical of the entire disease population; however, this limitation is mitigated by the fact that both of these institutions are large referral centers, so the studies included are likely to be representative of cases evaluated in a wide variety of geographic areas. Third, some physician and patient-reported assessments were missing, which is expected in a retrospective study. Fourth, specific clinical measures of axial disease activity were not available as they are neither validated nor routinely collected in pediatric clinical care. Although these latter two issues limited the assessment of construct and discriminative validity, they do not affect the feasibility or reliability assessment. These relatively minor limitations are to be expected in the first systematic assessment of the feasibility and reliability of a new tool. Fifth, there was no gold standard by which to assess construct validity of the tool. The closest constructs to measure clinical (not radiographic) disease activity were the juvenile SpA disease activity index and the physician global assessment of disease activity. At most, we hypothesized there would be modest correlation. The absence a current gold standard should not preclude assessment of the feasibility and reliability utility of the tool.

## Conclusions

We have demonstrated the feasibility and reliability of the SPARCC sacroiliac joint inflammation scoring methodology in children with established or suspected spondyloarthritis. This scoring system is based upon dichotomous scoring of lesions on consecutive slices through the cartilaginous part of the sacroiliac joint. We have not only established feasibility but also demonstrated that inexperienced readers can have their reading calibrated using standardized definitions, DICOM reference cases and an interactive calibration module. Further work is needed to assess the responsiveness and prognostic significance of the SPARCC SIS in evaluating MRI lesions in children.

## Abbreviations

*ASAS*: Assessment of SpA International Society
CRP: C-reactive protein
*DICOM*: Digital Imaging and Communication in Medicine
*ICC*: Intraclass correlation coefficients
*JSpA*: Juvenile spondyloarthritis
*JSpADA*: Jjuvenile SpA disease activity index
*MRI*: magnetic resonance imaging
*SIS*: sacroiliac joint inflammation score
*SpA*: spondyloarthritis
*SPARCC*: Spondyloarthritis Research Consortium of Canada
*STIR*: Short-tau inversion recovery
*TNF*: Tumor necrosis factor
